# Urban regeneration as population health intervention: a health impact assessment in the Bay of Pasaia (Spain)

**DOI:** 10.1186/s12939-016-0424-7

**Published:** 2016-09-15

**Authors:** Elena Serrano, Isabel Larrañaga, Maite Morteruel, María Dolores Baixas de Ros, Mikel Basterrechea, Dolores Martinez, Elena Aldasoro, Amaia Bacigalupe

**Affiliations:** 1 Public Health and Addictions Division of Gipuzkoa, Regional Public Health Centre Bidasoa, Basque Government, Avd. Navarra, 41-20302 Irún, Spain; 2 Public Health and Addictions Division of Gipuzkoa, Basque Government, Avd. Navarra 4, 20013 San Sebastian, Spain; 3 BIODONOSTIA Health Research Institute, San Sebastian, Spain; 4 BIOEF Basque Institute of Health Research, Bilbao, Spain; 5 San Pedro Health Center, Osakidetza-Basque Public Health Service, c/Los Marinos 1, 20110 Pasaia, Spain; 6 Consortium for Biomedical Research in Epidemiology and Public Health (CIBER en Epidemiología y Salud Pública-CIBERESP), Barcelona, Spain; 7 Department of Environment and Regional Planning, Basque Government, c/Infanta Cristina. 11- Villa Begoña, 20008 San Sebastian, Gipuzkoa Spain; 8 Public Health and Addictions Directorate, Basque Government, Vitoria-Gasteiz, Spain; 9 Department of Sociology 2, University of the Basque Country (UPV/EHU), Leioa, Spain; 10 OPIK- Research Group Social Determinants of Health and Demographic Change, Leioa, Spain

**Keywords:** Health impact assessment, Urban regeneration, Social determinants of health, Health inequalities, Mixed method design

## Abstract

**Background:**

An important health issue in urban areas is how changes arising from the regeneration of city-areas affect social determinants of health and equity. This paper examines the impacts attributable to a new fish market and to delays in the regeneration of a port area in a deteriorated region of the Bay of Pasaia (Spain). Potential differential impacts on local residents and socially vulnerable groups were evaluated to determine health inequalities.

**Methods:**

An in-depth, prospective and concurrent Health-Impact-Assessment (HIA) focused on equity was conducted by the regional Public Health Department, following the Merseyside guidelines. Data from different sources was triangulated and impacts were identified using qualitative and quantitative methods.

**Results:**

The intervention area is characterised by poor social, environmental, and health indicators. The distinctness of the two projects generates contrasting health and inequality impacts: generally positive for the new fish market and negative for the port area. The former creates recreational spaces and improves urban quality and social cohesion. By contrast, inaction and stagnation of the project in the port area perpetuates deterioration, a lack of safety, and poor health, as well as increased social frustration.

**Conclusions:**

In addition to assessing the health impacts of both projects this HIA promoted intersectoral partnerships, boosted a holistic and positive view of health and incorporated health and equity into the political discourse. Community-level participatory action enabled public health institutions to respond to new urban planning challenges and responsibilities in a more democratic manner.

## Background

All over the world urban populations are growing and urbanisation is one of today’s major public health challenges [1]. In 2014, more than 70 % of Europe’s population lived in cities [2]. This growth increases the risk of health inequalities due to the proliferation of deprived areas where infrastructure and services are insufficient to meet the needs of the residents. Multiple determinants, such as green areas, walkability, public transport, affordable housing, health and education services, employment, urban safety, and social cohesion converge to influence the health status and wellbeing of city dwellers [3]. The available evidence suggests that investing in urban renewal and acting on social determinants of health (SDH) may produce health benefits and reduce health inequalities [4, 5]. Some conceptual models help us understand the factors and processes operating in urban areas and influencing health [6, 7]. They advocate good governance and intersectoral action as requisites for tackling the root causes of health and inequity in cities.

**Fig. 1:**
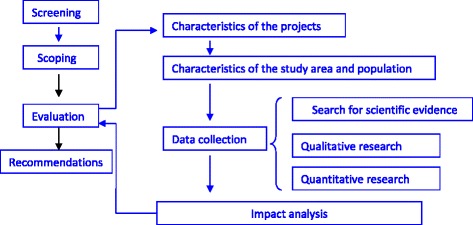
Sequence of steps of the HIA

In recent years Health Impact Assessment (HIA) has been proposed as an essential tool for addressing SDH and developing a Health in All Policies (HiAP) approach. It was launched in 2006 [8] as a governmental strategy for improving population health by coordinating action across health and non-health sectors. Among the variety of intersectoral initiatives used to implement HiAP, HIA encourages evidence-based decisions about political initiatives that shape social determinants of health, so that these policies result in the healthiest options. An HIA also determines the differential impacts of an intervention on both the general public and specific groups, and assesses whether such impacts are inequitable. Thus, HIAs promote the systematic consideration of health inequalities as part of political agendas and planning [9].

### HIA of the Bay of Pasaia

The present study describes an urban regeneration HIA in the Bay of Pasaia, a port area in the province of Gipuzkoa, northern Spain. Between 1960 and 1970, increased port activity led to a marked population increase and the growth of working-class neighbourhoods around the bay. These were characterised by a high population density, environmental degradation, and a lack of services. Decreased port activity in the 1980s was followed by social, economic, and urban deterioration, and successive revitalisation proposals were repeatedly postponed. The latest Master Plan for the comprehensive regeneration of the bay was presented in 2010. It outlined progressive urban redevelopment and contained several subprojects, centred on the construction of an outer port. The environmental and economic costs of the new port were highly controversial and the plan was rejected by many sectors of the population. Furthermore, due to the economic and political upheaval that followed the recent economic downturn, only two of the subprojects, the new fish market (NFM) and the redevelopment of the La Herrera (LH) zone, achieved a certain degree of concretion.

The NFM project proposed constructing a wholesale fish market in the town centre, including a new recreational area. The LH project was designed to redevelop a disused and degraded area, located in the town centre next to the NFM. Progress in the LH project was impeded by disagreements between the two sponsor institutions, City Hall (CH) and the Port Authority (PA). This project was halted and no alternative solution approved. The lack of action was accompanied by progressive deterioration of the LH plot and complaints from the population. Focus was subsequently shifted to the effects of delaying the LH project.

A HIA focusing on equity was then conducted to assess the health impacts of both the NFM project and the postponement of the LH project. The aim was to issue recommendations and promote informed and effective decision-making regarding public health. The HIA was part of the Basque Government’s Department of Health policy [10], which promotes a Health in All Policies (HiAP) strategy in all decision making.

This article describes the process and outcomes of the HIA on regeneration projects in the bay of Pasaia, and discusses the main difficulties, opportunities and lessons learned that may be relevant to future urban regeneration HIAs.

## Methods

An in-depth, prospective and concurrent HIA focused on equity was conducted from 2012–2013. The HIA team comprised professionals from different backgrounds (public health, environmental and regional planning, and GPs), and was led by the regional Public Health Department.

The HIA was conducted according the Merseyside guidelines [11], which provide the main organisational stages for a HIA (Fig. 1):

1. Screening: a checklist proposed by the Devon Health Forum [12] was used to determine the appropriateness and feasibility of the HIA. It made a preliminary assessment of positive and negative effects on health and health inequalities, as well as areas of uncertainty and potential improvements. Interest in and acceptance of the HIA by the decision-makers (sponsor institutions) was also considered.
2. Scoping: this involved defining the evaluation reference terms including the affected populations, data and methods, participation and involvement of stakeholder and decision-makers. Disagreements between the sponsor institutions (PA and CH) prevented the formation of a single Steering Committee. To overcome this, parallel meetings were held with the two sponsors to agree on the scope of the HIA and report and discuss the results and final recommendations.
3. Evaluation: data from various sources was triangulated to fully define the characteristics of the study population, the nature of the projects, their differential effects on socially vulnerable groups, the public’s perception of them and their impact. Table 1 details the information sources and methods used in this stage, which included:
  - *Characteristics of the projects:* based on a desktop review and meetings between the HIA team and technical staff overseeing the projects.
  - *Characterisation of the study area and population: a* comprehensive baseline review of data on health and health-related determinants was undertaken. Demographic, socioeconomic, urban, and environmental factors that influence health and health equity were assessed using the available data, existing reports, and through direct observation of the area by the HIA team (Table 2).
  - *Search for scientific evidence*: information was gathered from the literature relating to the effects of regeneration plans on health and SDH as identified in the screening, such as urban quality (recreational areas, parks, and footpaths), accessibility, security, transportation (traffic, noise, and contamination), employment, economic dynamism, social cohesion, and collective self-esteem. Tables 4 and 5 show the references reviewed for the main SDH.
  - A mixed method involving both qualitative and quantitative research was applied to obtain detailed knowledge of the public perception of the projects, and the main impacts on health and health inequalities.
    Firstly, stakeholder and community perspectives were gathered qualitatively (18 in-depth interviews and 5 focus groups). In addition, to promote community participation in the evaluation process by giving an active voice to players in aspects of municipal life, this approach allowed people to give their views on the projects and impacts, and suggest potential improvements. It also provided data on the relationships between the socio-historical, urban, and health-related dimensions. Sessions were recorded and transcribed upon consent. Analysis was performed from a sociological discourse analysis perspective. More details of the qualitative study have already been published [13].
    Secondly, quantitative data was gathered to gauge the magnitude of the problems and impacts and complement the results of the qualitative study (Table 3). Three hundred three residents selected from the telephone directory using quota sampling according to deprivation in the area of residence, age and sex, were interviewed by phone. The quotas were proportional to these variables in the population. Residence census tracts were categorised into 2 groups according to the deprivation index [14] (least deprived: deprivation index within quintiles I-III vs most deprived: deprivation index within quintiles IV-V); age was also grouped into 2 categories (18–44 vs 45–79 years). Telephone numbers were grouped by deprivation index of the corresponding census tract and selected by random-digit dialling until each quota was complete. Information on impacts, community support for recommendations and their potential effects on lifestyle, and people-place attachment, was collected using a structured questionnaire. Verbal informed consent was obtained from all participants.
  - *Impact analysis:* the information collected was integrated into an *impact matrix* for each project, combining health effects related to each SDH, their distribution in the population, and the data sources used (qualitative study, quantitative study, and literature review) (Tables 4 and 5).
4. Recommendations: based on the analysis of the health and inequalities impact data collected, a set of recommendations were developed for mitigating potential negative impacts and enhancing potential positive impacts (Table 6).

**Table 1:** Sources of information used and methods applied in the evaluation process

Evaluation phase	Evaluation method	Study population	Study contents
a) Project characteristics	Review of the technical documentation provided by the sponsor institutions	LH and NFM projects	Analysis of projects: design, location, target population and other affected groups, effects on social inequalities, implementation schedule.
	Interviews with architects of the Master Plan and managing engineers	LH and NFM projects	
b) Characterisation of the study area and population	Socio-demographic records	Population of the study area	Sex, age, origin, education level, deprivation index, relation to activity.
	Health records: mortality, cancer, hospital discharges, primary care records Basque Health Survey	Population of the study area	Health status of the population, chronic diseases. Health habits of the population
	Environmental records: air, noise, and soil pollution	Study area	Contamination levels: PM_10_ particles in air, ambient noise, degraded terrain.
	Urban quality data	Study area	Population density, green spaces
	Direct observation by HIA team	Study area and plots for LH and NFM. Urban dynamics	Physical characteristics of the area Initial state of the plots and their surroundings Person-space relationship: places of significance
c) Search for scientific evidence	Review of scientific literature: - Health-specific sources: Medline, Embase, The Cochrane Collaboration, Campbell Collaboration, Health Evidence Network - Multidisciplinary sources: Web of Knowledge, SCOPUS - HIA-specific portals: CREIS, HIA Gateway, WHO, e IMPACT-Health Impact Assessment International Consortium	Publications, studies, reviews, documents, reports from similar HIAs	Search for evidence on the following SDH: - urban quality: recreational areas, green spaces, footpaths, walkability - safety - transport and accessibility: access to services, nearby traffic, noise and pollution associated with traffic - employment and economic dynamism - social networks, social cohesion and collective self-esteem
d) Mixed methods: Qualitative and quantitative research	Stakeholder and community group perspective: qualitative study - In-depth interviews	*N* = 18 qualified participants (representatives of associations, neighbourhood organisations, health professionals, town planners)	Identification of: - interrelationships between socio-historical, urban, and health-related dimensions in the context of the studied projects - public perception of the project effects on the urban environment and health/quality of life - health inequality-related issues Channelling the participation of affected populations in the assessment process. Collection of proposals for potential improvements
	- Focus groups Data analysis: sociological discourse analysis	*N* = 5 groups, stratified according to age, social class, and activity (youths, housewives, adults, senior citizens)	
	Magnitude of problems and impacts: quantitative study - Quota sampling by deprivation index of the census tract of residence, sex, and age - Telephone survey - Analysis: descriptive, inter-group comparison, Chi square test	*N* = 303 residents	Identification and quantification of: - deficits and problems in the area - places of significance, attachment to environment and social identity - potential effects of improved urban quality on lifestyle and collective self-esteem - social inequalities according to sex, age and socioeconomic status

**Table 2:** Socio-demographic, environmental and health baseline profile of the study area

Socio-demographic characteristics	Study area			Gipuzkoa		
- Inhabitants	20,862			705,210		
- > = 65 years (%)	22.3			19.8		
- Unemployment rate (%)	12.9			10.1		
- College education (%)	15.6			23.3		
- Foreign-born residents (%)	8.0			6.5		
Environmental characteristics	Study area			Standard values		
- Housing density (dwelling/ha)	130.5			60.6		
- Green spaces (%)	10.5			20.1		
- PM_10_: high peaks (μg/m^3^)	48–228			50 (daily average)		
- Noise levels daytime/night-time (dB(A))	10 dB(A) higher than standard values			65/55		
- Degraded land (ha)	11			--		
	Study area			Gipuzkoa		
Health status	Men	Women	Total	Men	Women	Total
Morbidity						
- All cancers (rates × 100^3^ inhabitants. Age-adjusted to European population)^a^	972.1**	403.9**	639.01**	513.03	275.9	377.6
- Chronic obstructive pulmonary disease (cases/100 IHC)^b^	2.87**	1.15**	1.99**	1.74	0.82	1.61
- Diabetes mellitus (cases/100 IHC)^b^	6.59**	6.45**	6.51**	5.37	4.23	4.79
- Arterial hypertension (cases/100 IHC)^b^	17.94**	20.54**	19.28**	15.90	16.38	16.17
- Anxiety-depression (cases/100 IHC)^b^	5.18**	14.18**	10.05**	3.64	8.82	6.27
Risk factors^c^						
- Obesity (%)	15.6	15.3	15.4*	14	11.9	12.9
- Smoking (%)	31.8	26*	28.9*	28	19.3	23.5
- Sedentary lifestyle (%)	57.5	57.2	57.3*	45.3	53.2	49.4
All-causes of mortality (rates × 100^3^ inhabitants). Age-adjusted to European population)^d^	860.6**	354.6	567*	694.7	353.8	502.6
Consumption of psychotropic drugs (DDD)^e^	-	-	75.6**	-	-	51.9
Hospital admissions (rates × 100^3^ inhabitants). Age-adjusted to standard European population)^f^	1203.3**	1019.8**	1086.2**	995.3	913.7	940.4

*p < 0.05; **p < 0.01

Source: ^a^Gipuzkoa Cancer Registry (1995–2004); ^b^Osakidetza stratification database (2011); IHC = Individual Health Card; ^c^ESCAV Health Survey of the Basque Country (2007); ^d^Mortality Registry of the Basque Country (2004–2008); ^e^Pharmacy Registry, DDD = Defined Daily Dose; ^f^ CMBD Hospital Discharge Register (2005–2009)

**Table 3:** Assessment of the area’s problems and the effects of the improvements

Problems in the area	Total % (CI-95 %)	Deprivation level^a^			Sex			Age		
		High	Low	*p*	Men	Women	*p*	18–44	45–79	*p*
Lack of recreational areas	68.7 % (63.4–73.9)	60 %	72 %	0.03	69.1 %	68.2 %	N S	70.8 %	66.4 %	NS
Walking difficulty	37.5 % (31.9–43.0)	37 %	37.8 %	NS	32.1 %	43 %	0.05	38.6 %	36.4 %	NS
Use of metro	39.3 % (33.7–44.7)	28 %	46 %	0.003	38 %	40.5 %	NS	40.4 %	38.1 %	NS
Lack of emblematic locations	35.3 % (29.9–40.7)	30 %	38 %	NS	30 %	40.5 %	0.05	32.7 %	38.1 %	NS
Potential effect attributed to recommended improvement: pedestrian walkway										
Increases physical activity	78.4 % (73.7–83.1)	73 %	81 %	0.06	75 %	81.7 %	NS	84 %	72.4 %	0.01
Increases use of the metro	64 % (58.5–69)	56 %	68 %	0.02	63.4 %	64.5 %	NS	70.6 %	56.9 %	0.01
Improves sociability	81.6 % (77.2–86)	87 %	79 %	NS	79.6 %	83.6 %	NS	86.5 %	76.4 %	0.01
Increases leisure opportunities	63.5 % (58.1–69)	56 %	67 %	0.04	61.6 %	65.4 %	NS	67.1 %	59.7 %	NS
Increases attractiveness of area	90.2 % (86.9–93.6)	98 %	86 %	0.001	91.1 %	89.4 %	NS	92.9 %	87.3 %	NS

^a^High deprivation level: includes residents from census tracts with lower deprivation index (quintile I-III); and low deprivation level: residents from census tracts with higher deprivation index (quintile IV, V)

**Table 4:** Impact matrix for NFM

Intervention phenomena	Structural and proximal determinants affected	Vulnerable groups and social inequalities in health	Potential health effects	Source of evidence
Characteristics of the new fish market (walkable roof garden and emblematic building)	*Green spaces and recreational areas* (+) ↑ physical activity (+) ↑ unstructured activities and social interaction *Pedestrian walkways* (+) ↑ physical activity (+) ↑ efficiency of land use (+) ↑ access to services and employment *Urban quality* (+) ↑ physical activity (+) ↑ diet quality (+) ↑*Social cohesion* (+) ↑ *Individual and social self-esteem* (+) ↑Employment and social dynamism	Positive effects on the population in the area next to the fish market, in particular those who are unemployed and/or have low incomes, no car, and cyclists, pedestrians, women, children, and the elderly	(+) ↑ wellbeing and quality of life (+) ↓ poor mental health (+) ↑ self-esteem (+) ↓ stress and fatigue (+) ↑ perceived physical health (+) ↑ sleep quality (+) ↓ chronic diseases: cardiovascular disease, diabetes, arterial hypertension, obesity and others (+) ↓cancer (+) ↑musculoskeletal health (+) ↓premature mortality	Qualitative study Quantitative study Literature review: [34–46]
Operation and activity of the fish market	*Environmental quality* (−) ↑ Noise, odours, persistence of pollution *Persistence of heavy traffic* (−) ↑Accident rate	Negative effects on the entire population, especially in urban areas close to the market and the associated access roads. Increased risk of accidents for children, youths, and the elderly	(−) ↓ mental health, ↑stress and irritability (−) ↑ cognitive disorders in children (−) ↑ cardiovascular disease, cancer, mortality due to diabetes mellitus and other causes, and exacerbation of COPD and asthma (−) ↑ injuries, accident-related disabilities	Qualitative study Literature review:[47–52]

(+) Positive impact; (−) Negative impact; ↑ Increase; ↓ Decrease

**Table 5:** Impact matrix for La Herrera

Effects of non-intervention	Affected social determinants of health	Vulnerable groups and social inequalities in health	Potential effects on health and bibliographical sources	
Persistence of degraded area	*Deteriorating physical environment* (−) ↓ physical activity (−) ↓ active transport (−) ↓ social cohesion and collective self-esteem *Perception of insecurity* (−) ↓ physical activity (−) ↓ social cohesion (−) ↓ employment and economic activity	Negative effects on the entire population, especially women, the elderly, children, and those with low incomes	(−) ↑ obesity, DM II, hypertension, and cancer (−) ↓ musculoskeletal health (−) ↓ perceived physical and mental health (−) ↑ depression and anxiety (−) ↓ sleep quality (−) ↓ self-esteem (−) ↑ stigma and psychosocial stress	Qualitative study Quantitative study Literature review:[36, 37, 41, 44, 46, 53–57]
Underfunding of potential uses of the area	*Mixed use of land and recreational areas* (−) ↓ active transport (−) ↓ social interaction	Negative effects on the entire population, particularly pedestrians and those with low incomes	(−) ↑obesity (−) ↓mental health and wellbeing	Qualitative study Quantitative study Literature review:[58, 59]
Unsafe and unequal access to metro	*Accessibility, public transport and connectivity* (−) ↓ physical activity (−) ↓ access to services (−) ↑isolation and ↓social cohesion (−) ↑contamination, noise and accidents due to increased traffic	Negative effects on the entire population, particularly women, children, the elderly, ethnic minorities, the disabled, and those with low incomes	(−) ↑ obesity and chronic diseases (−) ↓ mental health (−) ↑ cardiovascular disease, exacerbation of COPD and asthma, cancer, mortality (−) ↑ irritability, stress and sleep disorders	Qualitative study Quantitative study Literature review:[37, 47, 48, 60]
Worsening of conflict with sponsor institutions	*Psychosocial sphere* (−) ↑ mistrust of institutions (−) ↑ sense of social frustration (−) ↓ sense of belonging to the community, collective identity and self-esteem	Negative effects on the entire population, particularly those who are socially excluded.	(−) ↓ physical and mental health	Qualitative study Literature review:[61, 62]

(−) Negative impact; ↑ Increase; ↓ Decrease

**Table 6:** Recommendations

NFM recommendations	
Design a route for heavy vehicles to the market via the port road, outside the urban centre.	
Establish a speed limit for road traffic and lay noise-absorbing asphalt along the route for heavy vehicles to the market in order to minimise noise and emissions.	
Establish a heavy vehicle parking area in the port area so that trucks waiting to load or unload in the market do not saturate the town’s parking areas.	
Establish regulations to ensure that engines of vehicles parked in loading/unloading areas are switched off, thus minimising noise and emissions.	
Provide soundproofing and particle filtering systems for ventilation systems located along the NFM’s walkable roof garden.	
Provide sufficient adequate lighting for the walkable roof garden, avoiding discomfort to the residents of the nearby houses, particularly those located at the same level as the walkable roof.	
Recommendations in response to delays in the LH project	
Prioritise regeneration of this area by planning for mixed land use, based on key deficits in the area (insufficient recreational areas, green spaces and parking areas).	
Involve the affected population in the decision-making process and keep them informed of the resolutions taken.	
Remove piles of demolition debris from the plot and clean and sanitise the area.	
Disinfect and apply pest control measures to buildings in the area, pave the plot and maintain the fence in an appropriate condition.	
New opportunities linked to the projects: pedestrian walkway	
Create a pedestrian walkway from the metro station to the mouth of the harbour, along the water’s edge.	
Provide new outdoor recreational areas including equipment that promotes physical activity and social relationships, applying the criteria of accessibility for all.	
Adequately illuminate the pedestrian walkway to minimise light pollution.	
Create green spaces, applying economic and environmental sustainability criteria: non-invasive, non-allergenic species with non-costly maintenance.	

## Results

This section presents the results of the evaluation and the recommendations (third and fourth stages described in the method). It includes an analysis of the projects, population characteristics, results of qualitative and quantitative research, impact matrix for each project, and recommended improvements.

### Characteristics of the projects

The scheduled timeframe for the NFM project was 2011–2014. The project proposed a landmark building with a walkable roof garden overlooking the bay. The building was designed to occupy the site of the old fish market, a 4-hectare plot in the urban centre, concealing the commercial activity of the market while still requiring trucks to pass through the town’s main street.

The LH project proposed new residential and service-related uses for a much deteriorated 7-hectare plot, located in the town centre close to the NFM site. The project did not obtain municipal licensing due to disagreements between the sponsor institutions regarding the proposed future uses of the plot. The project did not obtain municipal licensing due to disagreements between the sponsor institutions regarding the proposed future uses of the plot. The PA mainly proposes new housing development whereas the CH proposes community infrastructure and recreation areas. As a consequence the regeneration project was halted and the plot in the town centre remained degraded and unsafe.

### Characterisation of the study area and population

In 2012, the study area had 20,862 inhabitants. Compared with the average for the Gipuzkoa province, the population was significantly older, had a higher unemployment rate, was less educated, and had a higher proportion of foreign-born residents. The study area was economically deprived, with 89.0 % of the census tracts within the three lowest deprivation index quintiles. It also had a higher housing density than the average for the province, as well as a lower percentage of green spaces (Table 2).

Environmental problems persisted despite gradual improvements. In 2013, the concentration of PM_10_ met the regulations, but 6 % of measurements detected high peaks (48–228 μg/m^3^). Peaks in daytime and night-time noise levels of more than 10 dB (A) above the reference limits were also recorded. The urban centre contained about 11 hectares of degraded land (Table 2).

A health analysis for the area revealed higher mortality rates, increased incidence of cancer, and a higher prevalence of chronic diseases (chronic obstructive pulmonary disease, diabetes mellitus, hypertension, obesity, and anxiety-depression) than in Gipuzkoa in general. A higher consumption of anxiolytic and antidepressant drugs was observed, along with more hospital admissions than the average for the province. Unhealthy habits were more prevalent, with higher smoking and physical inactivity rates (Table 2).﻿

### Stakeholder and community group perspectives

Gathered through qualitative research. The general public’s perception of the regeneration projects’ health impacts pivoted around a biomedical understanding of health. A strong awareness of deficits in social endowments and a sense of institutional neglect dominated the discourse, often overlapping with issues more directly related to health. Political dissension between the two institutions and the resulting impact on the regeneration process (delays and area degradation), as well as a lack of information and transparency were all mentioned by participants.

A certain degree of heterogeneity and complementarity of views according to social status was identified. Participants with higher socioeconomic status had a more technical-political perspective, focusing on macro aspects of life in society, while participants with the lowest socioeconomic status focused more on everyday problems.

In addition to the importance per se of these factors and their influence beyond the areas of intervention, focus groups and in-depth interviews identified impacts associated with the projects. The NFM was generally perceived as enhancing the urban quality of the area, given the symbolic nature of the building, the creation of new recreational areas, and the associated improvements in walkability. Improvements in social cohesion and job creation as well as greater economic dynamism were also identified as potential positive effects associated with the creation of new, quality spaces. The NFM’s location in the town centre and goods vehicles associated with its commercial activity were perceived as factors that would increase the accident risk in addition to noise, odours, and contamination.

The stagnation of the LH project resulted in outrage and frustration over repeatedly unfulfilled promises and the perpetuation of a deteriorated environment. The main concerns were related to unsatisfactory urban maintenance, poor health, insecurity, and a lack of recreational areas. These frustrations, expressed by the majority of citizens, had a negative impact on collective self-esteem, hindering the adoption of healthy habits such as physical activity or social interaction.

The study participants also proposed further improvements, such as a pedestrian walkway along the bay that would connect the port with the metro station. This proposal called for an urban axis to integrate the NFM and LH, support civic activity, and enhance the urban quality of the area.

### Quantitative magnitude of the problems and impacts

A total of 303 people were surveyed with an average age of 47.6; 49 % were aged between 18 and 44 and 51 % were over 45. Women comprised 50.5 % of the sample group.

Table 3 lists the most important problems reported according to respondents’ age, sex, and residential area deprivation index. Over two thirds cited a lack of recreational areas, just as identified in the qualitative study. This proportion was higher in residents from census tracts with greater social deprivation. Walking difficulties attributable to the environment were mentioned by 37.5 %, a complaint that was significantly more common among women. Participants identified the following causes for this: a lack of pedestrian walkways and areas appropriate for walking (76 %); hilly terrain (48 %); and excessive traffic (47 %). Use of the metro was scarce; 61 % of participants had never used it and 29.3 % only occasionally. Only 10 % were regular users (≥3 days a week). The proportion of users was significantly higher among residents of deprived areas. Access to the metro was deemed poorly illuminated (87 %), unsafe (85 %), poorly maintained (85 %), and dirty (83 %).

The pedestrian walkway proposed in the qualitative phase was valued very positively given its potential to improve urban quality. The assessment was more positive among younger respondents and residents of the most deprived areas, who viewed the walkway as a means of increasing physical activity, facilitating use of the metro, providing more social and leisure opportunities, and enhancing the area’s attractiveness (Table 3).

The findings showed limited appropriation of and attachment to the public space among respondents. Of those surveyed, 35.0 % could not identify a place of special value and subjective significance in the area, a response that was more common among women.

### Impact analysis

Tables 4 and 5 summarise the data from the evaluation process. They show the affected SDH, health impacts, and health inequalities identified from the literature review and qualitative/quantitative studies. The impacts of each of these elements were rated according to their positive or negative effects on health and health inequalities.

### Recommendations

Issued in two stages: (i) after the screening phase, before construction of the NFM began, in order to minimise impacts during the building work; and (ii) after analysing the evaluation process, based on the participants’ requests and the potential improvements identified.

The recommendations were grouped into three categories focused on: a) improving the impact of the NFM project; b) minimising the negative effects of the delays and inaction affecting the LH; and c) new opportunities for improvement, particularly the pedestrian walkway due to both its expected effect on the SDH and the community support it received (Table 6).

The final report containing the results of the evaluation and the resulting recommendations [15] was presented and discussed with the sponsors, stakeholders, and citizens’ associations involved in the HIA, who broadly agreed with the proposals. At the end of 2015, according to the recommendations of the HIA, an institutional agreement was reached to build the pedestrian walkway bordering the bay between the metro station, the walkable roof garden of the new fish market, and the harbour mouth [16]. To implement this, a budget was approved and a timeline was established that envisaged the work would be completed by 2018. The other recommendations were partially implemented and will be assessed once the urbanisation of the fish market is complete.

## Discussion

### Main findings

The assessed projects were part of a comprehensive regeneration plan, the complexity of which was conditioned by the historical and political context of the affected community [17]. The intervention area was characterised by poor social and health indicators, and had experienced prolonged environmental and urban deterioration, further accentuating its overall deprivation.

The differing nature of the two projects, as well as their development, resulted in opposing health and health inequality impacts; overall, these were positive for the NFM and negative for LH. The NFM project responded to a degraded urban environment with few recreational or social areas and poor walkability, contributing to improved urban quality and social cohesion. The lack of intervention in the case of LH had a negative impact as it perpetuated the deterioration, insecurity, poor health, and social frustration. Fourteen recommendations were proposed for improving the projects, their health effects and their distribution within the population. These have been partially accepted, and their compliance will be evaluated at the end of the projects.

Beyond effectiveness in implementing recommendations, this HIA produced other relevant outcomes, in terms of changing the views and attitudes of community participants and decision-makers, raising awareness of health issues, and promoting intersectoral partnerships. The HIA therefore functioned not only as a decision-making tool for urban planning but also as a way to construct a practical framework for supporting and developing the HiAP strategy, by integrating health considerations into decisions made outside the health sector [18].

### HIA and the challenge of complexity

Regeneration processes are complex with varied and diffuse impacts; these can influence and be influenced by contextual factors [19]. Addressing this complexity requires skilled communication, participatory action, searching for evidence, and various data collection methods.

In this HIA, data obtained through the qualitative study helped explain the situation of conflict, low collective self-esteem, and social pessimism. Moreover, these findings revealed the importance of the area’s historical background, with repeated rehabilitation proposals that were never realised due to a lack of institutional consensus, generating feelings of abandonment in the population. According to urban psychology [20], environmental qualities help shape individual and collective identity through the phenomenon of space appropriation. Dilapidated and unsafe environments negatively affect social esteem, as was evident in the study population. This fact could explain the higher prevalence of anxiety and depression and the increased consumption of psychotropic drugs in Pasaia as compared to the rest of the province.

The findings also highlighted characteristics of the regeneration processes that should be considered during the evaluation. One such example is the labile and evolving nature of the projects, with frequent changes and delays in their development, having collateral effects through differential perceptions of the proposed time frame [17, 21]. Thus, delays that may be perceived as reasonable at the institutional level are unacceptable to the affected population, exacerbating unrest and conflict.

The lack of information and transparency in project management was another source of mistrust. These deficiencies are common in young democracies with little experience of public participation in decision-making, as described in other urban regeneration HIAs in Spain [22, 23]. In our case, disseminating information relating to the projects during the HIA process contributed to improving the public’s perception of transparency.

The evaluation process also revealed that identifying the project’s potential health impacts presented a challenge for some of the participants. Similar difficulties have been described in relation to HIA, due to the general public’s predominantly biomedical perspective [24]. However, the participatory process articulated through the qualitative research helped provide a more social perspective of health, linking it to both health- and non-health-related public policies. Finally, it is worth noting the level of public participation and the restorative effect this had on the self-esteem of the people of Pasaia, who, although aware of the advisory nature of the study, assumed an active role in the process, and felt their opinions were valued.

The quantitative study assessed the importance of social and urban problems in the area and their relevance to different population subgroups. People positively valued the incorporation of quality urban spaces, given that these promote sociability, leisure, exercise, and walkability, and related health improvements. Moreover, the population’s broad endorsement of this feature and its likely promotion of healthy behaviour in the most disadvantaged social groups underlined the value of these measures as mechanisms for generating equity.

The lack of urban landmarks or areas of special significance likely to generate some form of place attachment was one of the key findings. The fact that more than one third of the respondents had difficulty identifying a place of subjective significance in the area emphasises the importance of this deficit and the need for regeneration projects. Some studies show that spaces which can create a sense of attachment or establish emotional links have a positive effect on the formation of identity, the sense of community, and emotional and social wellbeing [25, 26]. There is also strong evidence that social links and ties within a community increase the chances of improving and maintaining the community’s health, even in adverse conditions [27]. This fact becomes extremely interesting in communities like the one studied here, with indicators of fragmentation and social conflict that lead to the deterioration of health and welfare [28].

### Strengths and limitations of the HIA

Although equity is a key principle in an HIA, its comprehensive introduction into the analysis often poses a challenge, given that it adds complexity [29]. One of the strengths of the present study was the explicit incorporation of an equity perspective across the entire process. Potential inequality axes were analysed when characterising the population, and different population groups potentially affected were considered in each stage (screening, literature review, qualitative and quantitative assessments, impact matrices, and recommendations).

An important aspect of the HIA is its flexibility: it evaluates not only the effects of proposed projects, but also the impact of inaction and delays in project implementation. Non-intervention is generally regarded as a control situation, necessary to identify the effects of an intervention in the short or medium term [17]. In our case, the lack of action and delays in the LH project constituted findings with significant adverse effects for the study population.

Urban redevelopment projects may promote place attachment and increase a community’s physical and psychosocial capital [25]. Including these issues in the assessment as relevant determinants of health was one of the most important contributions of this HIA. In this way the HIA may be a mediating tool for catalysing policies based on the capabilities of individual communities and designed to generate health assets and resources [30].

The use of multiple data sources and the triangulation of qualitative and quantitative data is another strength of this HIA. While a HIA is a context-sensitive tool, in-depth understanding of context requires different methods [31]. The application of qualitative and quantitative research was particularly useful for addressing the complexity of the study area, as this provided a broad view of relevant historical and political mechanisms, and shed light on the public’s perspective of the intervention as well as the dimension of the problems and impacts. In the conflictive context of the study, the mixed method was also aimed at improving the HIA’s credibility [31].

This HIA has additional limitations that, although not affecting the overall results, should be taken into consideration. The division of competences between institutions governed by different political parties further complicated the HIA process, hindering consensus dynamics. The institutional confrontation made it impossible to bring together all of the sponsors in a single HIA steering committee, and necessitated parallel briefings and discussions with the two main institutions (PA and CH). Another limitation is that the HIA cannot be separated from the context in which it was undertaken. Spain has little tradition of intersectoral collaboration and participation in decision-making processes [32]. Despite the introduction of HIA into national legislation [33], a lack of policy development has limited the effectiveness of the process in applying the recommendations.

The limited sample size and design of the quantitative research could also be a weakness of this study. Although, a larger sample would have provided more statistically reliable and powerful estimates, the surveyed population was considered adequate for statistical analysis considering the descriptive nature of the research and the available resources. Moreover, quota sampling did not ensure the representativeness of the sample, but it did provide information on key groups and variables for this HIA, such as social disadvantage, age and gender.

Despite these limitations, the HIA process was positive as it helped identify impacts, informed decision-makers, integrated health and health equity considerations into urban planning, and placed value on intersectoral partnerships. It therefore aided in putting the HiAP strategy into practice, which is one of the Basque Government’s 2013–2020 Health Plan objectives [10]. Moreover, this experience may represent a milestone that adds to the knowledge and expertise necessary for achieving such goals.

## Conclusions

The differing context, nature, and implementation of the two projects generate contrasting health and inequality impacts: generally positive for the NFM and negative for LH. The HIA, as a structured framework for participatory action at community level, allows public health institutions to respond to new urban planning challenges and responsibilities in a more democratic manner. The present evaluation process promoted a holistic and positive vision of health, incorporating health and equity into the political discourse. It provided a platform for the communication and negotiation necessary in the context of high levels of institutional-community conflict and dissension, which often accompanies urban redevelopment projects. In addition to a framework based on values of equity and dialogue, this HIA facilitated partnerships between sectors, institutions and citizens and fostered transparency and public accountability.

## Abbreviations

CH: City Hall
COPD: Chronic obstructive pulmonary disease
DM: ﻿Diabetes Mellitus
HIA: Health impact assessment
HiAP: Health in all in all policies
LH: La Herrera
NFM: New fish market
PA: Port Authority
PM_10_: Particulate matter less than 10 μm
SDH: Social determinants of health
